# Multi-Environmental Trials Reveal Genetic Plasticity of Oat Agronomic Traits Associated With Climate Variable Changes

**DOI:** 10.3389/fpls.2018.01358

**Published:** 2018-09-19

**Authors:** Nicolas Rispail, Gracia Montilla-Bascón, Javier Sánchez-Martín, Fernando Flores, Catherine Howarth, Tim Langdon, Diego Rubiales, Elena Prats

**Affiliations:** ^1^Institute for Sustainable Agriculture, CSIC, Córdoba, Spain; ^2^ETSI La Rábida, University of Huelva, Palos de la Frontera, Spain; ^3^Institute of Biological, Environmental and Rural Sciences, University of Aberystwyth, Aberystwyth, United Kingdom

**Keywords:** agronomic traits, climate variables, genetic plasticity, genome wide association studies, oat

## Abstract

Although oat cultivation around the Mediterranean basin is steadily increasing, its yield in these regions lags far behind those of Northern Europe. This results mainly from the poor adaptation of current oat cultivars to Mediterranean environments. Local landraces may act as reservoirs of favorable traits that could contribute to increase oat resilience in this region. To aid selection of suitable agro-climate adapted genotypes we integrated genome-wide association approaches with analysis of field assessed phenotypes of genetic variants and of the weight of associated markers across different environmental variables. Association models accounting for oat population structure were applied on either arithmetic means or best linear unbiased prediction (BLUPs) to ensure robust identification of associations with the agronomic traits evaluated. The meta-analysis of the six joint environments (mega-environment) identified several markers associated with several agronomic traits and crown rust severity. Five of these associated markers were located within expressed genes. These associations were only mildly influenced by climatic variables indicating that these markers are good candidates to improve the genetic potential of oat under Mediterranean conditions. The models also highlighted several marker-trait associations, strongly affected by particular climatic variables including high rain pre- or post-heading dates and high temperatures, revealing strong potential for oat adaptation to specific agro-climatic conditions. These results will contribute to increase oat resilience for particular climatic conditions and facilitate breeding for plant adaptation to a wider range of climatic conditions in the current scenario of climate change.

## Introduction

Oat (*Avena sativa* L.) is a major cereal and fodder crop grown worldwide over approximately 9 million hectares. A major division of the crop is into white oats (*A. sativa* subsp. *sativa* L.), used for human consumption and fodder, and preferred for milling, and red oats (*A. sativa* subsp. *byzantina* (K. Koch) Romero Zarco) preferred for forage (Stevens et al., 2004). In the last two decades the oat cultivated area steadily increased within the Mediterranean rim, at a rate of 7500 ha/year (FAO, 2015). Thus, the cultivated area in Northern and Southern Europe is currently very similar with approximately 1 million hectares each. However, the yield of northern regions is 2.7-fold higher than that of Mediterranean areas. This highlights the need to improve oat yield by increasing oat resilience to Mediterranean conditions.

High temperatures and drought are among the most yield limiting factors for oat cultivation under the Mediterranean climate (Coffman, 1955). In addition, diseases can result in important yield losses (Prats et al., 2015). In particular, crown rust (*Puccinia coronata*) causes high losses in yield and grain quality worldwide (Simons, 1985) but especially in the Mediterranean Basin where rust populations are more virulent than in the north and center of Europe (Herrmann and Roderick, 1996). The lack of adaptability to the south of oat cultivars bred in northern regions may be alleviated by the inclusion of better-adapted Southern oat landraces in oat breeding programs. Landraces are good reservoirs of favorable traits related to stress tolerance and resilience to the Mediterranean environment. For instance, oat landraces have demonstrated similar yield and higher rust resistance than oat cultivars under Mediterranean conditions (Sánchez-Martín et al., 2017).

The challenge is, then, in selecting genotypes better adapted to Mediterranean agro-climatic conditions. This is not straight-forward as they are highly affected by genotype × environment interactions. Indeed, oat landraces have shown higher variation among different environments than commercial cultivars (Sánchez-Martín et al., 2017). The use of genetic markers may ease the selection of genotypes with particular desired characters, as it has been shown for several species during the last decades. In oats, bi-parental populations have allowed the identification of quantitative trait loci (QTL) linked to key agronomic traits, such as flowering and heading dates, vernalization, and stress resistance and quality traits, such as tocopherol, oil, and protein content of seeds (Holland et al., 2002; Wooten et al., 2009; Jackson et al., 2010; Hizbai et al., 2012; Nava et al., 2012; He et al., 2013). However, the progenitors of these bi-parental populations are in most cases not representative of the overall pool of genotypes readily available for breeding. In addition, the transfer of the QTL-associated markers into different genetic backgrounds is not always possible limiting their implementation in marker-assisted selection (MAS) (Snowdon and Friedt, 2004).

Association analysis has been shown to circumvent these limitations leading to the uncovering of novel markers associated with desirable traits such as favorable agronomic, stress resistances, or quality traits. In oats, association studies have provided molecular markers for yield, adaptation, resistance to biotic and abiotic stresses such as rust and powdery mildew infection, lodging and quality traits such as beta-glucan concentration (Achleitner et al., 2008; Newell et al., 2012; Montilla-Bascón et al., 2015; Winkler et al., 2016; Tumino et al., 2017). Association studies rely on linkage disequilibrium (LD), which is the non-random association of alleles at two loci (Falconer and MacKay, 1996). LD is related to reproductive biology and breeding history (Flint-Garcia et al., 2003; Newell et al., 2011) Association analysis takes thus advantage of ancestral recombination events that have occurred within a sample of individuals to detect correlations between genotypes and phenotypes within these individuals (Zondervan and Cardon, 2004). Genome-wide association studies (GWAS) can potentially identify the genetic polymorphisms responsible for important traits. The power of association studies lies in the degree of LD between the marker allele and the functional allele responsible for the trait under study. In oats, LD between markers has been demonstrated to be extensive; in particular, association studies for disease resistance under controlled conditions with the oat collection described below demonstrated a high LD, indicating high genome coverage with non-associated markers (Montilla-Bascón et al., 2015). However, LD is affected by population structure, selection, mutation, drift, and admixture (Flint-Garcia et al., 2003; Newell et al., 2011). This may led to spurious associations and the identification of false positives (Abiola et al., 2003; Zhang et al., 2010). Thus, despite its high potential as an efficient and valuable gene discovery tool, GWAS have to contemplate the presence of population structure to limit the detection of spurious marker-trait associations. Different statistical approaches performed on the oat collection used in the current study revealed a moderate population structure involving four clusters (Montilla-Bascón et al., 2015). Each of these clusters contained a reasonably high genetic diversity, equivalent to approximately 10% of the diversity seen in the representative panel used for global oat germplasm characterisation (Tinker et al., 2009). The level of diversity plays a critical role in the identification of significant marker-trait associations (Ingvarsson and Street, 2011). Several statistical models have been developed to account for strong population structure in order to limit the number of false-positives (Price et al., 2006; Kang et al., 2008). However, suitable models for a particular population and trait combination may not apply to other situations. The phenotypic estimate methods [arithmetic means, best linear unbiased predictors (BLUPs)] should also be carefully chosen since they could affect model outcomes. Thus, the fitting of the models has to be scrupulously examined for each situation to ensure the validity of the result obtained.

Climate change will affect crop performance and alter the range of phenotypes that a single genotype can express. Environmental effects are expected to be particularly significant for complex agronomic traits such as yield, which are influenced by multiple genetic components. Even within a specific region of cultivation, such as the Mediterranean rim, the role of specific genomic regions may be differentially influenced by particular climate variables, which must be accounted for by appropriate weighting of models. Understanding these variables will help to breed more effectively for improved resilience to new and unstable climate scenarios. In oats, no studies have attempted to identify markers associated with improved oat performance under Mediterranean conditions. Furthermore, no association studies have attempted to associate markers with the effects of climate variables on oat agronomic traits.

In the present study, we aimed to identify genetic markers significantly associated with important agronomic traits, and crown rust resistance, in oat cultivated under Mediterranean conditions. An oat collection consisting of 177 accessions, including 141 Mediterranean landraces, and 36 European commercial cultivars with high genetic diversity has previously been shown to be useful for association studies (Montilla-Bascón et al., 2015). We tested the oat collection in three different locations during two different seasons with a total of six environments in order not only to detect markers significantly associated for the mega-environment (ME) but also to identify markers explaining a high percentage of the phenotypic variation associated with particular variations of climate variables. This will facilitate breeding for plant adaptation to a wider range of climatic conditions.

## Materials and Methods

### Plant Material

The oat panel used consisted of 177 accessions. As stated in Montilla-Bascón et al. (2015), the panel comprised, “141 white and red oat landraces, provided by Centro de Recursos Fitogenéticos, INIA, Madrid, and 36 commercial varieties supplied by the Andalusian Network of Agriculture Experimentation (RAEA). The commercial oat varieties were: Ac1, Acebeda, Adamo, Aintree, Alcudia, Anchuela, Araceli, Brawi, Caleche, Cannele, Chambord, Chappline, Charming, Cobeña, Condor, Cory, Edelprinz, Flega, Fringante, Fuwi, Hammel, Kankan, Kantora, Karmela, Kassandra, Kazmina, Mirabel, Mojacar, Norly, Orblanche, Pallini, Patones, Prevision, Primula, Rappidena, and Saia. A list of the landraces evaluated together with other relevant data from gene bank and the genetic relationships between the oat accessions has been previously reported (Montilla-Bascón et al., 2013).”

### Genotyping and Phenotyping of the Oat Collection

Genotyping of the collection was performed by Diversity Arrays P/L, using the high density oat array (15,000 markers) as described in Tinker et al. (2009). Additional SSRs were used as described in Montilla-Bascón et al. (2013). From a total of 2086 polymorphic alleles identified, 11 markers that showed a call rate lower than 80% and 56 that showed a MAF < 0.01 were removed. A total of 476 redundant markers were also merged into 169 groups representing these markers (**Supplementary Table S1**). Following data curation a total of 1712 markers were used for association purposes.

The collection was evaluated over two crop seasons at three contrasting locations in Spain, at Córdoba, Escacena, and Salamanca. The combination of each location and year constitute a particular environment whereas we considered the locality as the specific site evaluated over the 2 years of evaluation. Sánchez-Martín et al. (2017) demonstrated that all environments in this study belong to the same ME; the term is used here to refer to average conditions. “At each location, a randomized complete block design with three replicates was used. Each replicate consisted in independent plots consisting of three 1-m-long rows bordered by the rust-susceptible oat cultivar Cory with the aim of providing the most appropriate conditions for the disease development. Within each plot, the rows were separated from each other by 30 cm, at a sowing density of around 90 seeds m^-2^. Sowings took place between November and December, according to local practices, except in Córdoba during the season 2010–2011 in which, due to intense rain levels, the sowing took place in January (**Supplementary Figure S1**). No irrigation or artificial inoculation were performed at any location, crown rust infection occurring naturally. Hand weeding was carried out when required, and no herbicides or fertilizers were applied. Trials were hand-harvested” (Sánchez-Martín et al., 2017).

Details of phenotyping of agronomic traits including grain yield, biomass, harvest index (HI), heading date (measured as growing degree days to panicle emergence, GDD), and crown rust severity together with a description of the genotype by environment (GxE) interactions have been reported in Sánchez-Martín et al. (2017).

### Association Study

Marker-phenotype associations were estimated with the software package TASSEL 4.1.27 (Bradbury et al., 2007). Initially, five models comprising general linear models (GLM) and mixed linear models (MLM) were tested using the ME data. Out of these five models, the two models which overall better accounted for the population structure of the oat collection and minimized the number of false positives were retained and applied to each trait and environment/locality/ME analysis. These were a GLM model using PCAs covariates and a MLM using both the percentages of admixture and the kinship coefficients as cofactors (Q and K matrices). Models were run using both mean data and best linear unbiased prediction (BLUPs) for the estimation of random effects and to determine the best fitting of the models.

General linear models procedures tested fixed-effect models in which mean phenotypes of a given trait were predicted by the independent variables. Tests were run with 1000 permutations allowing determination of the site-wise *p* value for each marker, which is the probability of a greater *F* value under the null hypothesis that the polymorphic loci is unrelated to the phenotype. To assess the correct fitting of the data and the control for type I error, a combined quantile-quantile plot that compare the observed distribution of –log10(*P*) values for each marker-trait association with the cumulative was drawn for all models and methods. Selection of the optimal model was performed by estimating the genetic inflation factor λ_median_, as follow:

$$\begin{array}{c} \lambda_{\mathrm{median}}\;=\;\{\mathrm{median}(S_{c\;+\;1},S_{c\;+\;2},...,S_{n})/0.456\}. \end{array}$$

A genetic inflation factor higher than 1.25 (λ > 1.25) indicates data stratification even after structure corrections. Therefore all models with λ > 1.25 were discarded and were not included in subsequent meta-analysis to avoid false positives (Reed et al., 2015).

The critical *p* values for assessing the significance of associations were corrected for multiple comparisons based on the false discovery rate (FDR) criteria (Benjamini and Hochberg, 1995), restricting the cut-off value for each model to limit the number of false positives below 1 (Newell et al., 2012). The matrix of *p* value was used to estimate the corresponding *q* values with the QVALUE package (Storey, 2002) in R (R Core Team, 2017).

### Non-metric Multidimensional Scaling Ordination and Canonical Correspondence Analysis

Ten climate variables [maximum temperature, minimum temperature and accumulated rain (mm) during pre-heading, at heading and post-heading period, and humidity] were obtained from the AEMET,^1^ Junta de Andalucía^2^ and Junta de Castilla León databases^3^ for each location (**Supplementary Figure S2**). Humidity refers to mean percentage of relative humidity during the growing season. To evaluate the influence of environmental factors on the significant markers, data were subjected to non-metric multidimensional scaling ordination (NMDS) (Anderson, 2001). This ordination technique is well-suited to handle non-normal and non-continuous data (Anderson, 2001) allowing the reduction of the climate variable matrix before modeling each trait. Canonical Correspondence Analysis (CCA) is a multivariate method that allows elucidation of the relationship between a factor (biological individual, marker, or trait) with the environment extracting synthetic environmental gradients from the datasets. These gradients are the basis to visualize the relationship between the factor and the environments through ordination diagrams offering additional information about the particular environmental variables that influence the behavior of the factor. CCA was used to establish the effect of the climatic estimates on the phenotypes and on the percentage of explained phenotypic variance of each marker. CCA was computed on PAST software (Hammer et al., 2001). Venn diagrams were produced with web-based tool InteractiVenn^4^ (Heberle et al., 2015).

### Annotation of Associated Markers

Although most associated markers are expected to be non-functional, the DArT marker sequencing data was used for annotation using BLAST (Altschul et al., 1990) searches to the nr and EST databases of Genbank (Data of release Oct, 30 2017 15:49 P.M.) with the BLASTn function. Matches were considered significant when e-value and % identity was higher than 1e^-20^ and 75%, respectively.

## Results

As described previously in Montilla-Bascón et al. (2015), the accessions used in this study clustered into four population groups: “the first cluster included mainly the white commercial varieties, cluster 2 the red oats, cluster 3 the white oat landraces characteristic to high altitude locations, and cluster 4 the white oat landraces more adapted to low altitude” (Montilla-Bascón et al., 2015). The oat collection showed high LD with approximately 300,000 marker pairs with *r*^2^ < 0.1, which allowed uncovering strong association in previous studies (Montilla-Bascón et al., 2015). A detailed GxE interaction study that included the phenotyping of the oat collection (Sánchez-Martín et al., 2014, 2017) confirmed that, for all assessed traits, the different environments were included within the same ME (subset of similar environments).

### Fitting of the Models

Correct fitting of the models is crucial to obtain reliable phenotype-marker associations. Preliminarily, we tested several models with the data including: a simple GLM, a GLM model using the percentages of admixture of each accession (Q matrix) as cofactors to take population structure into account (GLM-Q), a GLM model using the PCAs covariates as cofactors (GLM-PCA), a GLM model using both Q matrix and PCAs covariates (GLM-Q-PCA) and a MLM using both the percentages of admixture and the kinship coefficients as cofactors (Q and K matrices). In addition, we ran the models using both the means and the BLUPs. Out of all models tested, the GLM-PCA and the MLM models yielded the lowest overall genetic inflation factors and were chosen for further analysis. **Table 1** shows the λ values obtained with these two models for each environment, locality (different environment from the same site), and ME. Most models showed a genetic inflation value very close to 1 suggesting appropriate adjustment for potential substructure. Models that did not fulfill the expectation were discarded from the analysis. **Supplementary Figure S3** depicts as an example the distribution of *p* values obtained with these models for rust severity. As stated, the degree of deviation from the red solid line representing the null expectation (absence of type I error) measured formally by the λ-statistic (Devlin et al., 2001) was very small for most models. Visual inspection of the depicted models and calculation of λ values lead us to conclude that for several traits the use of both GLM and MLM with either arithmetic means or BLUPs estimates was appropriate.

**Table 1:** Genetic inflation factor (*λ*) for each of the models tested for association.

Lambda	Co09^∗^	Co10	Co	Es09	Es10	Es	Sa09	Sa10	Sa	ME
**RUST**								
GM	1.14	1.16	1.19	1.33	1.23	1.33	1.15	1.04	1.12	1.26
GB	1.11	1.07	1.18	1.28	1.12	1.31	1.14	n.e	n.e.	1.16
MM	1.11	1.07	0.98	1.12	0.87	1.00	1.07	0.96	1.06	1.25
MB	0.98	1.03	0.98	1.15	0.86	0.97	1.08	n.e	n.e.	1.14
**YIELD**								
GM	0.98	1.22	1.00	1.31	1.31	1.32	1.09	1.28	1.29	1.29
GB	1.02	1.08	1.04	1.25	1.25	1.25	1.08	1.25	1.31	1.29
MM	0.94	1.12	0.97	0.92	0.92	1.11	1.01	0.93	0.97	1.01
MB	0.99	1.02	0.91	0.90	0.90	1.06	1.03	0.94	0.98	1.03
**Flowering (GDD)**								
GM	1.40	1.12	1.19	1.32	1.65	1.53	1.17	1.89	1.86	1.47
GB	1.27	1.12	1.18	1.05	1.55	1.35	0.97	1.78	1.86	1.50
MM	1.04	0.90	0.92	1.06	0.01	1.03	0.95	1.25	1.14	1.11
MB	1.03	0.89	0.93	0.95	1.05	1.04	0.89	1.43	1.14	1.16
**BIOMASS**								
GM	0.99	1.13	1.00	1.20	1.00	1.08	1.09	2.05	1.24	1.08
GB	1.06	1.08	0.99	1.21	0.99	1.07	1.09	1.96	1.25	1.07
MM	0.94	0.94	0.91	0.98	0.95	0.99	1.00	1.29	0.95	0.97
MB	0.98	0.96	0.91	0.94	0.96	0.97	1.00	1.25	0.95	0.97
**HI**								
GM	1.26	1.32	1.36	1.26	1.23	1.30	1.17	1.55	1.49	1.62
GB	1.11	1.20	1.36	1.13	1.10	1.29	1.11	1.25	1.56	1.62
MM	0.92	1.10	0.95	1.12	1.02	1.10	1.00	1.06	0.94	1.08
MB	1.02	1.09	0.96	1.02	0.99	1.12	1.08	0.97	0.96	1.04

*^∗^Co, Es, and Sa indicate the localities of Córdoba, Escacena, and Salamanca, respectively. The numbers following the localities indicate the year of the trial: 2009 (09) and 2010 (10). GM and GB, General Linear Model (GLM) accounting for population structure according to principal component covariates, PCA, using mean values (GM) or best linear unbiased predictors (BLUPs) (GB); MM, MB, Mixed Linear Model, (MLM), corrected with kinship and structure matrices using mean values (MM) or BLUPs (MB). Due to distribution of data for disease severity in Sa10 with high numbers of zero values, BLUPs-based models were not estimated (n.e.).*

### Weight of Particular Environments on Marker Significance

**FIGURE 1:**
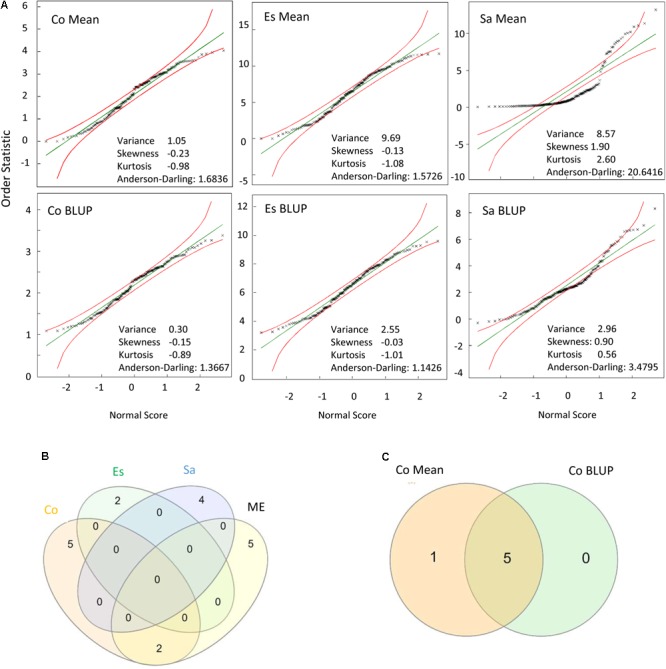
**(A)** Normal Q–Q Plot for crown rust severity in the localities tested: Córdoba (Co), Escacena (Es), and Salamanca (Sa) depicting 95% confidence limits for adjustment to normality. Data for analysis were recorded during two consecutive seasons at each locality. **(B)** Venn diagram indicating the number of significant markers identified in each locality and in the mega-environment. **(C)** Venn diagram indicating significant markers for rust severity between models considering means or BLUPs estimates at Córdoba.

**FIGURE 2:**
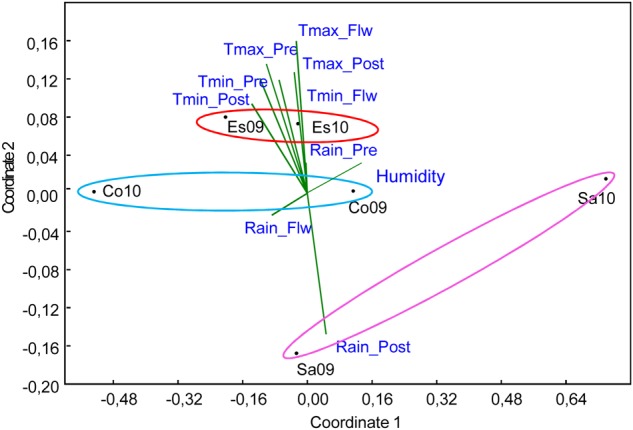
Non metric multidimensional scaling (NMDS) analysis of nine climate variables including: maximum temperature (Tmax), minimum temperature (Tmin) and rain during different growing stages [pre-flowering (pre), flowering (Flw), and post-flowering (post)] characterizing the six environments, which are the combination of three localities: Córdoba (Co), Escacena (Es), and Salamanca (Sa) and 2 years 2009 (09) and 2010 (10), used for phenotyping. Environment sharing the same locality were grouped with ovals of different colors.

To uncover the biological significance of the identified marker-trait associations, the association study was performed not only for the joint data (ME) but also for each environment and locality. Interestingly, some significantly associated markers in a particular environment were also significantly associated in the ME while many others were not. In addition, the association study from the ME data reveal novel significant association not detected for any of the single environments or localities. The former occurred when a marker was highly significant in a particular environment but not significant in the others while the latter occured when a marker was nearly significant in most single environments. We observed that the phenotypic data distribution is key for the strength of association but is not the only parameter to take into account. Thus, when phenotypic data were represented in a Q–Q plot (**Figure 1A**) and data had a good fit with the assumed normal distribution, confirmed for instance by low values of the Anderson-Darling statistic (as observed for Córdoba and Escacena data), markers were associated with higher probabilities and had a higher weight in the analysis of the ME. However, the representativeness of the particular environment on the average environment, which can be derived from heritability-adjusted genotype plus G×E (HA-GGE) biplot analysis (i.e., Sánchez-Martín et al., 2017) and NMDS analysis as in **Figure 2** also played a crucial role in the strength of association for the ME. Thus, significant association detected in environments/localities closer to the ME average, as observed for instance for Córdoba, with environments near the vector origin (**Figure 2**), were more likely to appear significant in the meta-analysis. For instance two out of the seven markers significantly associated with crown rust severity in Córdoba, were also highly significant in the ME whereas none of the significant markers from Escacena or Salamanca were significantly associated with crown rust severity in the ME (**Figure 1B**, see **Supplementary Figure S4** for examples in other traits). Regarding the type of estimates used in the models, data showed that significant markers derived from BLUPs-based models were also significantly associated with the trait in means-based models. However, although BLUPs improved the adjustment of data to normality, they greatly reduced the variance, which usually reduced the number of significantly associated markers (**Figure 1C**).

### Associations of Markers With Agronomic Traits

The meta-analysis of the six joint environments for association of the main agronomic traits with genomic regions highlighted several significant markers (**Table 2**). We found a total of five markers associated with yield, three with biomass, four with HI, five with heading date and seven associated with rust severity, respectively. Interestingly, marker Opt-8261 located onto Mrg23 (He et al., 2013) was significant for both yield and HI. As the sequence of most significant DArT markers has been released (Tinker et al., 2009), we compare them with the sequences from Genbank (nr database) (**Table 3**). As expected, most associated markers had no homology to any known gene confirming that the majority of the significantly associated markers are not located within the causative gene. However, significant homology to previously identified genes was detected for a handful of associated markers (**Table 3**). For example, oPt-10734 (accession number FI158728), located on Mrg05 (Hizbai et al., 2012), and highlighted as significantly associated with yield, matched with an iron/phytosiderophore transporter of *A. sativa*. oPt-12924 (accession number FI157582), located on Mrg12, matched with a cinnamoyl-CoA reductase 1 of *Aegilops tauschii.* oPt-8261 (accession number FI158468), which was associated for both yield and HI had some similarity to a *Hordeum vulgare* RNA sequence encoding the 30S ribosomic S17 protein. oPt-15271 marker (accession number FI157794) which is included in marker Merge 82, associated with heading date, matched with a sequence encoding a predicted non-specific lipid transfer protein. Interestingly, marker oPt-11494 (accession number FI159112), located on Mrg01 and associated with rust severity, matched to a nucleotide-binding site leucine-rich repeat (NBS-LRR) disease resistance protein homologue (rga S-226) of *Hordeum vulgare.*

**Table 2:** Markers associated with different agronomic traits in the mega-environment (composed of three sites evaluated over 2 years) according the GLM corrected for population structure according to principal component covariates, PCA, and Mixed Linear Model, (MLM), corrected with kinship and structure matrices and that in addition fulfill the criteria of a genetic inflation factor lower than 1.25. Marker-trait association was considered significant when the corresponding markers’ *q* value was lower than the q value cut-off that retrieve less than one false positive estimated for each model tested by false discovery rate test.

Marker	marker_p	markerR^2^	Map location	ref
**Yield**				
oPt-10734	0.00020351	0.06405	Mrg05	Hizbai et al. (2012)
oPt-12924	0.000035844	0.07632	Mrg12	He et al. (2013)
oPt-5217	0.00025632	0.06187	Mrg03	Tinker et al. (2009)
oPt-8261	0.00042453	0.05877	Mrg23	He et al. (2013)
oPt-9844	0.00033705	0.05992	NA	
**Biomass**				
Merge98	0.00049472	0.04305	NA	
oPt-15938	0.00077951	0.05059	Mrg15	Tinker et al. (2009)
**Harvest Index**				
Barb2-40	0.00055462	0.08744	NA	
MAMA07	0.00040226	0.07294	Mrg13	Wight et al. (2010)
oPt-793146	0.00046858	0.08982	NA	
oPt-8261	0.0005373	0.08783	Mrg23	He et al. (2013)
**Heading date**				
MAMA07	0.00034825	0.07102	Mrg13	Wight et al. (2010)
Merge82	0.00017619	0.09703	NA	
oPt-10359	0.000015774	0.12598	Mrg12	Tinker et al. (2009)
oPt-10891	0.000027511	0.11923	Mrg08	He et al. (2013)
oPt-1340	0.00055509	0.08356	Mrg08	He et al. (2013)
**Crown rust severity**				
Merge136	0.00031113	0.02328	Mrg18	Howarth (pers com)
oPt-10121	0.00097244	0.02484	Mrg21	Hizbai et al. (2012)
oPt-11494	0.0006268	0.02634	Mrg01	Hizbai et al. (2012)
oPt-12898	0.00057288	0.09602	NA	
oPt-388855	0.00034875	0.10272	NA	
oPt-794313	0.00015813	0.03099	NA	
oPt-9546	0.00113	0.02434	Mrg18	Howarth (pers com)

**Table 3:** Potential homologous sequence of significant markers using the function Blastn of the BLAST algorithm (Altschul et al., 1990). Only associated markers with significant matches with genes present in the database are listed.

Marker	Blastn	Species	E-value	Cov (%)	Ident (%)	Accession Number
oPt-10734	Iron/phytosiderophore transporter	*Avena sativa*	0.0	100	92	FJ477297.1
oPt-12924	cinnamoyl-CoA reductase 1	*Aegilops tauschii* subsp. *tauschii*	5E-60	28	89	XM_020303167.1
oPt-8261	EST DK805720 seedling shoot and root in salt treatment or abscission library	*Hordeum vulgare*	5E-21	20	81	DK805720.1
oPt-15271	PREDICTED: non-specific lipid-transfer protein-like protein At2g13820	*Aegilops tauschii*	4E-49	33	91	XM_008667359.2
oPt-11494	NBS-LRR disease resistance protein homologue (rga S-226)	*Hordeum vulgare*	7E-58	67	77	AJ507091.1

Analysis of markers with significant GxE associations, revealed, a contrasting relationship between genotypes highlighted as high-yielding and stable in HA-GGE biplots indicating adaptation to high temperature and lower rain level i.e., 79, 122, 133 (Sánchez-Martín et al., 2017), and genotypes highlighted as lower yielding due to adaptation to high rain level and cooler temperatures. Several markers identified as relevant for yield such as oPt-9844 or oPt-5217 showed presence/absence variation associated with these variables. This connection and the fact that several markers were highlighted by the models as significant in a particular locality (gathering two environments), but not in others despite all environments belonged to the same ME, lead us to analyse further the effect of particular climate variables on the associated markers.

### Effect of Climate Variable on the Phenotypic Variance Explained by Markers

First, we performed an NMDS analysis in order to better understand the climate parameters contributing to different environments at the various locations. **Figure 2** shows that the most important variables (with longer vectors) defining the relevant agronomic traits were the maximum temperatures, mainly pre- and post-heading, the minimum temperatures during the whole season and the rain during the post-heading period. Escacena was characterised by relatively high temperatures during the whole season [with positive projections (depicted) over these vectors] and low levels of rain post-heading [with negative projections (non-depicted) on that vector]. Salamanca followed the opposite pattern with high post-heading levels of rain and the coldest temperatures, although Sa10 showed a milder climate. Córdoba showed average conditions, with most projections falling near to the vectors origin, compared with the other two sites, confirming that it was more representative of the average environment.

**FIGURE 3:**
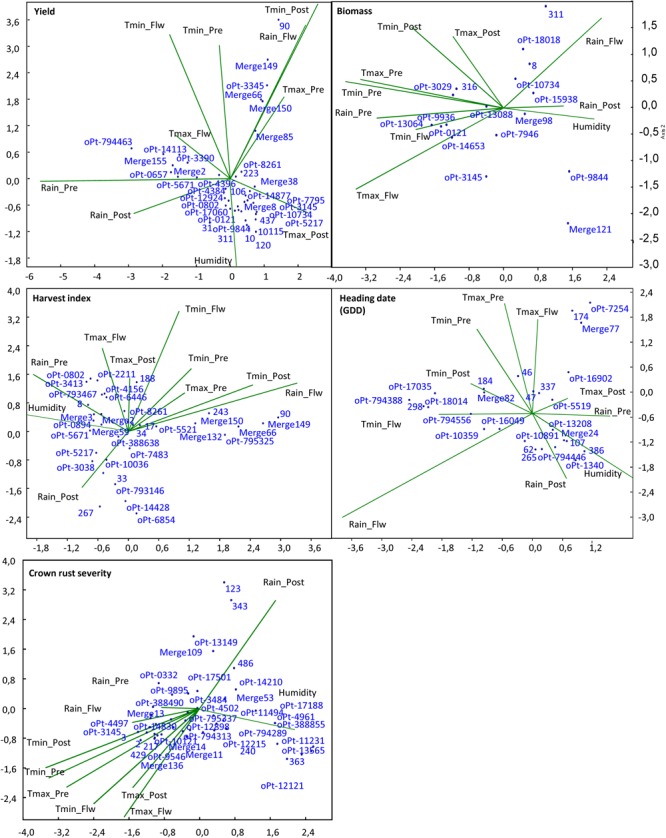
Canonical correspondence analysis of significant markers from the different environments and the influence of environmental factors in several agronomic traits. Tmax, maximum temperature; Tmin, minimum temperature; Pre, pre-flowering period; Flw, flowering period; Post, post-flowering period.

We then performed CCA in order to reveal the climate variable effect on the phenotypic variance explained by those markers that had been highlighted as significant in particular environments (**Supplementary Table S2**). **Figure 3** depicts the influence of the different climate variables on the phenotypic variance explained by significantly associated markers in the models. We observed that the phenotypic variance explained by the markers was highly influenced by climate variables, with many markers located in the extreme of the climate vectors. For yield, most markers, including the significant markers in the meta-analysis, grouped between the vectors of rain and maximum temperature post-heading, which are key factors determining yield (Sánchez-Martín et al., 2017). However, several markers explained a high proportion of the phenotypic variance under conditions of high temperature post-heading and rain during heading period, i.e., Merge 149, 90, oPt-3345, Merge 66, or Merge 150, while others are significantly associated with the trait in conditions of high rain pre-heading and temperatures during heading period, i.e., oPt-794463, Merge 155, oPt-0657, oPt-3390, and oPt-14113. Interestingly most of the former markers were significantly associated with yield in Co10 whereas most of the latter arose as significant in Co09 that showed these climatic characteristics (**Figure 2**). For biomass, markers highlighted as significant in the ME were near to the vector origin (i.e., oPt-15938 and Merge98) indicating the lowest influence of climate variable in them. However, other markers explained a higher proportion of the phenotypic variance under more extreme climate conditions such as high levels of rain (i.e., oPt-9844 or Merge 121) or higher temperatures (i.e., 316, oPt-3029). Interestingly, for crown rust severity, vectors related to temperatures fell opposite to rain post-heading vector confirming that moderate temperatures and humidity favor this disease. The plot shows that again most of the markers highlighted as significant in the ME (**Table 2**) tend to localize near to the vector origin suggesting that they were less influenced by particular climatic variables. By contrast several markers such as Merge 11, 2, 3, 429 123, and 343, or oPt-13149 explained higher phenotypic variance under higher temperatures and low rain level or vice-versa.

## Discussion

Nowadays, the usefulness of GWAS for marker-phenotype association in plants is widely recognized. However, previous studies on crop agronomic performance under field conditions have revealed great variability in applied methods used in terms of the models and their necessary correction, the type of data, the number of environments, and the probability test used to perform the association. In this work, we aimed to explore the influence of these parameters on the association analysis in order to find reliable markers for several agronomic traits in oat crop. The oat accession forming our oat panel was carefully selected to include diverse oat types, to achieve high genetic diversity. It includes white (generally spring sown) and red (generally autumn sown) oat types forecasting the existence of a population structure which have to be accounted. The collection contains approximately 10% of the polymorphic markers found in the global oat reference panel (Tinker et al., 2009) and was demonstrated to possess high LD (Montilla-Bascón et al., 2015) at a rate enabling detection of markers significantly associated with a trait (Ingvarsson and Street, 2011). Since the oat panel presented a moderate population structure, parameters such as Q and K matrices and PCA covariates were implemented to correct models for stratification. Nevertheless, since *A. sativa* and *A. byzantina* have significant genetic/chromosomal differences, with *byzantina* type originated from the wild *sterilis* type and the *sativa* type originated from the wild *fatua* type (Loskutov, 2008; Loskutov et al., 2011; Ladizinsky, 2012) and with genetic discrepancies reported by cytogenetics, FISH (Sanz et al., 2010; Badaeva et al., 2011) and morphological/genetic markers (Nikoloudakis et al., 2016), the identified markers might be correlated among taxa or “linked” to one of the two genotypic groups. To rule out this possibility, results were analyzed for each of the oat types separately. Removing data from red oat accessions did not significantly alter the overall conclusions of this study (data not shown) although the number of red oats used was not large enough to analyse them separately.

Optimal model fitting was confirmed both by depicting the normal probability plots and through genomic control (Devlin and Roeder, 1999; Reich and Goldstein, 2001; Zheng et al., 2006). Ideally, the observed markers *p* value should be distributed according to the null hypothesis of no marker-trait association except for a reduced number of markers that are in true association with the trait. However, potential genotyping errors, cryptic relatedness and/or the stratification of the population might distort artificially the marker allele frequencies leading to the inflation of the test statistics across the whole genome (Cardon and Palmer, 2003; Campbell et al., 2005). To determine whether a marker stand out from the analysis we used the genomic control method (Devlin and Roeder, 1999; Reich and Goldstein, 2001). In absence of population structure, association between unlinked markers and the trait should follow a χ^2^ distribution with 1 degree of freedom (Devlin and Roeder, 1999; Reich and Goldstein, 2001). However, when a population structure is present, this distribution is inflated by a value termed λ, that increase proportionally with sample size (Freedman et al., 2004). Yang et al. (2011) demonstrated that the analysis of quantitative trait with polygenic inheritance might also led to significant test statistic inflation even in absence of population stratification, which makes this genomic control necessary. Standard genomic control theory foresees that the expected value of λ_mean_ and λ_median_ are identical (Devlin and Roeder, 1999; Reich and Goldstein, 2001). However, for polygenic trait, λ_mean_ and λ_median_ follow different trends that vary according to the sample size, its heritability and its prevalence. We used λ_median_ since median provides a reliable estimate of the inflation factor even if a small fraction of the null loci actually affect liability to the trait or are linked to the gene under study (Devlin et al., 2001). This approach allowed us to reject those models in which high inflation factor did not assure reliable elimination of false positives. Models were run by using either means or BLUPs estimates, to determine their impact on the association analysis. Highly significant marker-trait associations were identified with both estimates while they do not significantly affect the genomic inflation. When data deviated from normality the use of BLUPs, improved the distribution considerably. However, it is well known that the use of BLUP reduces the variance of data even when predicted from a consistent model due to the prediction errors around the true values (Hadfield et al., 2010). This could be one reason explaining the lower number of significant marker-trait association revealed in BLUP-based models. Consequently, the use of BLUPs is advised only for data that do not fulfill the normality assumption.

We performed a systematic approach running the models for each of the localities (joint environments from the same site) in addition to the overall ME analysis (joint environments from all localities). This was done in order to find more reliable markers and to understand the relationship between the markers identified as significant in the ME and those identified in each locality. The rationale behind this was that better distribution of data in a particular site or data from a site with climatological conditions more similar to the average environment could influence the significance of the marker-trait association found in the meta-analysis (all joint environments). We observed that, as expected, under a high variance and optimal distribution of data, the significance of the marker-trait association was higher. In addition, associated markers highlighted as significant in those sites with environments that better reflected the average environment had a higher probability to be significant in the ME. On this basis, markers that arise in a particular site but not in the ME could be particularly influenced by the climatic variables of that site. To explore this possibility we performed NMDS and CCA (Ter braak, 1986). Interestingly, in most cases markers highlighted as significant in the ME, representative of the average environment, fall near the vector origin indicating that they were only mildly influenced by climatic variables. However, significant markers in particular sites explained a higher proportion of the phenotypic variance under particular climatic variables. This could be useful information in the context of climate change and to breed for adaptation to more extreme environmental conditions. Although, the proportion of the phenotypic variance explained by a marker in GWAS reflects the relevance of the markers in the variation of the traits it is necessary to take into account that very low *r*^2^ values are often observed in these studies. This is a consequence of so-called “missing heritability” (Maher, 2008; Manolio et al., 2009). This might be rooted to the fact that complex traits are often controlled by numerous genes each having only a little effect too small to be detected by the stringent significance criteria applied in GWAS (Manolio et al., 2009).

Previous studies have identified several QTL for the traits studied here. A dense hexaploid oat consensus map based on 12 bi-parental populations is now available (Chaffin et al., 2016) permitting more accurate comparative mapping between populations. Of the markers associated with heading date (GDD), oPt-10359 has been mapped onto Mrg12 (Tinker et al., 2009) near where previous studies have identified similar associations in a wide range of environments (Esvelt-Klos et al., 2016) and where Vrn 3 has been mapped (Nava et al., 2012). QTL on Mrg12 overlapping the marker-associations found here for heading date have also been found in a wide range of populations and environments (Siripoonwiwat et al., 1996; Holland et al., 1997, 2002; Tanhuanpaa et al., 2012). oPt 10891 and oPt 1340 are found 3cM apart in He et al. (2013) on Mrg08 near where QTL for flowering time have previously been found (Wooten et al., 2008). oPt 12924 was found in this study and also by He et al. (2013) to be associated with yield and maps close to oPt-10359 on Mrg 12. A further association with heading date was found here on Mrg13 in a similar location to a QTL for flowering time found previously (Barbosa-Neto et al., 2000; Wooten et al., 2009; Tumino et al., 2016).

Although most significantly associated markers were expected to be separate from the causative variation, five of them appear to encode for functional genes. Thus, marker oPt-10734 was identified as an iron/siderophore transporter. Interestingly, graminaceous plants, such as oats, use a chelation strategy as a mechanism for iron acquisition. These plants secrete phytosiderophores to solubilize soil iron, and uptaking the resulting iron-phytosiderophore complexes (Nozoye et al., 2011). Phytosiderophore secretion is crucial for plant growth and hence for yield (Shenker and Chen, 2005). Indeed, transgenic approaches to improve rice tolerance to low iron availability has been proposed as a practical way to increase crop production in adverse soils (Takahashi et al., 2001; Ishimaru et al., 2007). oPt-15271 with homology to a lipid-transfer protein was associated with heading date. A supporting role for lipid-transfer proteins during flowering has been described (Clark and Bohnert, 1999). Some members of this protein family are specifically expressed in the meristematic cell at the flowering and during embryo formation (Clark and Bohnert, 1999). Their role would be related to lipid secretion. Epidermal and sub-epidermal cell layers of the stigma are known to secrete a fluid particularly rich in lipids and phenolic compounds. These lipids might contribute to reduce the secretion evaporation being crucial for fecundation. Finally, it is significant that one of the markers associated with crown rust severity had high homology to a NBS-LRR disease resistance protein. Most of the disease resistance genes (R genes) cloned to date in plants encode NBS-LRR proteins characterized by nucleotide-binding site (NBS) and leucine-rich repeat (LRR) domains as well as variable amino- and carboxy-terminal domains. These large, abundant, proteins are involved in the detection of diverse pathogens, including bacteria, viruses, fungi, nematodes, insects and oomycetes (McHale et al., 2006). NBS-LRR proteins are involved in the recognition of specialized pathogen effectors [also called avirulence (Avr) proteins] that are thought to provide virulence function in the absence of the cognate R gene (Chisholm et al., 2006). Thus, oPt-10734, 15271, and 11494 are interesting candidate genes for further studies on yield, heading and rust resistance in oat, respectively.

The results provided here will allow the use of the genetic resources included in this study in breeding programs based on sound information of their adaptation to different environments and on the genetic regions more influenced by particular climatic variables. This knowledge may help to improve the oat crop in current but also future growing conditions.

## Author Contributions

NR conducted most of the bioinformatic analysis and modeling. GM-B and JS-M conducted most work related to genotyping and phenotyping, respectively. DR contributed to the stress resistance aspects. FF contributed to statistical aspect of the work. CH and TL contributed to genetic aspects. EP steered the research, designed experiments, and interpreted the results and drafted the manuscript. All authors participated in the critical reading and writing of the manuscript.

## Conflict of Interest Statement

The authors declare that the research was conducted in the absence of any commercial or financial relationships that could be construed as a potential conflict of interest.

## Footnotes

1 https://datosclima.es/Aemet2013/LocalizacionEstaciones.php

2 https://www.juntadeandalucia.es

3 https://datosabiertos.jcyl.es

4 http://www.interactivenn.net/
