# Supplementary material for: Multi-Environmental Trials Reveal Genetic Plasticity of Oat Agronomic Traits Associated With Climate Variable Changes
Source: Front Plant Sci. 2018 Sep 19;9:1358. doi: 10.3389/fpls.2018.01358 (PMC6156136; doi:10.3389/fpls.2018.01358)
Supplement: Supplementary file 1 [file Table_1.PDF]

Supplemental material

Multi-environmental trials reveal genetic plasticity of oat agronomic traits associated with climate variable changes

Nicolas Rispail[1], Gracia Montilla-Bascón[1], Javier Sánchez-Martín[1], Fernando Flores[2], Catherine Howarth[3], Tim Langdon[3], Diego Rubiales[1], Elena Prats[1*]

[1]CSIC-Institute for Sustainable Agriculture, Córdoba, Spain.
[2]E.T.S.I. La Rábida., Univ. Huelva, 21819 Palos de la Frontera, Spain.
[3]Institute of Biological, Environmental and Rural Sciences, University of Aberystwyth, UK

*Corresponding author: Elena Prats
elena.prats@ias.csic.es
tel: +34957499291

Supplemental Table 1: Redundant markers found in the analysis and the name of the groups in which these were merged.

Redundant markers	Number of markers	New marker name
oPt-6174,oPt-0185,9328,4014,8714, 5848,7285,3473,389221,1177,3249	11	Merge 001
oPt-7991+389252	2	Merge 002
oPt-6534+794382	2	Merge 003
oPt-11392+4947	2	Merge 004
oPt-1702+17861+17612+16941	4	Merge 005
oPt-388705+10644	2	Merge 006
oPt-794527, 17220, 2707, 6863, 2231	5	Merge 007
oPt-7732+11042+13919	3	Merge 008
oPt-14675+5738	2	Merge 009
oPt-7226+12932	2	Merge 010
oPt-7996+9423	2	Merge 011
oPt-17524+10163	2	Merge 012
oPt-7806+14536+10107+8247	4	Merge 013
oPt-11975+4506	2	Merge 014
oPt-17866+14172	2	Merge 015
oPt-3256+11190	2	Merge 016
oPt-16022+16110	2	Merge 017
oPt9944+11217+2660	3	Merge 018
oPt-3600+2785	2	Merge 019
oPt-17139+795330	2	Merge 020
oPt-14530+11056+5998+10313	4	Merge 021
oPt-4191+8899	2	Merge 022
oPt-0133+0151+11123	3	Merge 023
oPt-11559+17731	2	Merge 024
oPt-3706+8354+0477+14342	4	Merge 025
oPt-793246+388904+794573+10866+2203+14715	6	Merge 026
oPt-2647+8723+13837	3	Merge 027
oPt-8757+7954+3025	3	Merge 028
oPt-8120 + 7368	2	Merge 029
oPt-13230+12704	2	Merge 030
oPt-388679+1373	2	Merge 031
oPt-15383+14033	2	Merge 032
oPt-388846+13999	2	Merge 033
oPt-388909+388995	2	Merge 034
oPt-3804+13547	2	Merge 035

oPt-14984+5897+2019+13973+16660+0028	6	Merge 036
oPt-16408+16107+4084	3	Merge 037
oPt-16959+15098	2	Merge 038
oPt-3063+6710	2	Merge 039
oPt-3109+5343	2	Merge 040
oPt-7218+2230+6209	3	Merge 041
oPt-795573+793452	2	Merge 042
oPt-795443+795521+795819	3	Merge 043
oPt-5276+6270	2	Merge 044
oPt-3225+388659	2	Merge 045
oPt-10050+10224+1724	3	Merge 046
oPt-13461+8246+2744	3	Merge 047
oPt-17430+1661+8886	3	Merge 048
oPt-13205+2654	2	Merge 049
oPt-9634+3930	2	Merge 050
oPt-13167+15072	2	Merge 051
oPt-17672+17671	2	Merge 052
oPt-14525+14897+794705+17980	4	Merge 053
oPt-14574+11247	2	Merge 054
oPt-0428+14391+5423+7308	4	Merge 055
oPt-1424+9239	2	Merge 056
oPt-9929+4939	2	Merge 057
oPt-14857+9121+4689	3	Merge 058
oPt-795596+793141	2	Merge 059
oPt-388937+4308+9213	3	Merge 060
oPt-388943+oPt-1660+oPt-7693+oPt-9466+oPt-6092+oPt-7188+oPt-6437+oPt-4593+oPt-7143+oPt-3731+2670+5402+1357+8785+3558+oPt-3747+oPt-4470+oPt-2434+oPt-5574	19	Merge 061
oPt-5816+5065+1205+2377+8439+1269	6	Merge 062
oPt-389206+8378+9688+8804+388932+ 0573+1392+389179	8	Merge 063
oPt-0337+7413	2	Merge 064
oPt-11816+388715	2	Merge 065
oPt-15638+11599+17571	3	Merge 066
oPt-16012+2554	2	Merge 067
oPt795300+5188	2	Merge 068
oPt388969+2953	2	Merge 069
oPt-13153+4069+6877+388793+13620+12680 +1568	7	Merge 070
oPt-7652+13342	2	Merge 071
oPt-0932+11888	2	Merge 072
oPt-2070+2598	2	Merge 073
oPt-18263+15495	2	Merge 074
oPt-14615+17400	2	Merge 075
oPt-8509+1518	2	Merge 076

oPt-8724+6068	2	Merge 077
oPt-12177+10973+7113	3	Merge 078
oPt-15259+389172	2	Merge 079
oPt-8576+17867	2	Merge 080
oPt-794543+5851	2	Merge 081
oPt-15271+794318	2	Merge 082
oPt-8805+11363+7468+10473+3024	5	Merge 083
oPt-10962+6132	2	Merge 084
oPt-18107+1495	2	Merge 085
oPt-0625+4750+10190	3	Merge 086
oPt-11290+794507+794784+9940	4	Merge 087
oPt-16629+14987	2	Merge 088
oPt-4850+8827+5351	3	Merge 089
oPt-8691+2132+6060+12723	4	Merge 090
oPt-17611+1803+1881	3	Merge 091
oPt-14466+11406	2	Merge 092
oPt-9533+16936+15125	3	Merge 093
oPt-14251+5469+8478+7822	4	Merge 094
oPt-9307+13924	2	Merge 095
oPt-2195+15057	2	Merge 096
oPt-13157+4192+5695	3	Merge 097
oPt-5349+13901	2	Merge 098
oPt-12027+17858	2	Merge 099
oPt-15296+11639+3965+8846	4	Merge100
oPt-8186+5740	2	Merge101
oPt-793491+10651	2	Merge102
oPt-795252+6595+6774	3	Merge103
oPt-16349+16958	2	Merge104
oPt-3783+10708+10104	3	Merge105
oPt-4140+17840+1672	3	Merge106
oPt-793362+14677	2	Merge107
oPt-16165+16431	2	Merge108
oPt-389121+7417	2	Merge109
oPt-795673+5506	2	Merge110
oPt-7754+11134+1861+7580+15767+4537	6	Merge111
oPt-11232+13897+12861	3	Merge112
oPt-389223+3999	2	Merge113
oPt-389136+8259	2	Merge114
oPt-11779+11096+12166+11219+11734	5	Merge115
oPt-389103+9870	2	Merge116
oPt-794780+794864+795549+795665+793345+793339+0796+5937+9922+4972+389093+389011+388755+388505	14	Merge117
oPt-16369+15843+4810	3	Merge118
oPt-16112+1385	2	Merge119

oPt-0136+4498	2	Merge120
oPt-16563+8417+10395+6496+11229+5347+15310	7	Merge121
oPt-794642+795428	2	Merge122
oPt-15993+2056	2	Merge123
oPt-5185+6258+7136+17026	4	Merge124
oPt-8393+17469+14751+12706	4	Merge125
oPt-16095+9199	2	Merge126
oPt-15267+793326+0617	3	Merge127
oPt-793391+793353	2	Merge128
oPt-793280+793418+16939	3	Merge129
oPt-6749+388925	2	Merge130
oPt-11438+17710	2	Merge131
oPt-13202+389113	2	Merge132
oPt-14189+4225	2	Merge133
oPt-793426+793157+14469	3	Merge134
oPt-0988+4995	2	Merge135
oPt-12034+1652	2	Merge136
oPt-793206+795612+2907	3	Merge137
oPt-794399+1813	2	Merge138
oPt-16220+5562	2	Merge139
oPt-3127+17802	2	Merge140
oPt-795018+793236+5764	3	Merge141
oPt0504+10232	2	Merge142
oPt-16414+17759	2	Merge143
oPt-1075+16151	2	Merge144
oPt-17697+10074	2	Merge145
oPt-1284+1459	2	Merge146
oPt-794821+6935	2	Merge147
oPt-16678+8771	2	Merge148
oPt-8392+3882	2	Merge149
oPt-13517+15755	2	Merge150
oPt-11020+1176	2	Merge151
oPt-13347+8916	2	Merge152
oPt-793191+15986	2	Merge153
ot-15508+0052	2	Merge154
oPt-388912+14444	2	Merge155
oPt-11466+4875	2	Merge156
oPt-0373+4457	2	Merge157
ot-13262+9990	2	Merge158
oPt-3986+14468	2	Merge159
oPt-16745+6636	2	Merge160
oPt-2477+13953+2523	3	Merge161
oPt-10725+14958	2	Merge162
oPt-16721+10737+10620	3	Merge163
oPt-11721+11886	2	Merge164

oPt-795737+794604+10683	3	Merge165
oPt795423+3599	2	Merge166
oPt-5242+794340	2	Merge167
oPt-794664+794630+7139+0572	4	Merge168
oPt-17511+15018	2	Merge169

Supplemental Table 2: Percentage of phenotypic variance explained in the individual environments by those markers identified as significant in the analysis of the different sites.

Marker	Co09	Co10	Es09	Es10	Sa09	Sa10
			YIELD			
Barb2-40	0,01556	0,00276	0,0058	0,10006	0,02348	0,04431
AME105	0,02527	0,00181	0,00575	5,56E-04	0,03903	0,03486
HvXan	0,00218	0,10941	0,00199	5,59E-04	0,00462	1,14E-05
MAMA1	0,01118	7,06E-04	0,0468	0,00393	0,00186	0,00373
AM01	0,00393	0,00527	1,89E-04	0,06633	0,00124	0,01855
AM87	0,0106	0,01095	6,88E-04	8,64E-04	0,01144	0,02286
AM102	0,00779	3,44E-04	0,01182	0,00997	0,07802	0,00257
AM04	0,00148	0,00698	0,00456	0,01223	9,35E-04	0,04682
Merge126	0,00251	0,01138	0,00554	0,01278	0,00644	0,02149
Merge13	0,03295	0,01181	0,05557	0,0564	0,00171	0,00339
Merge149	0,00965	0,10975	0,00145	8,24E-04	0,00747	0,02026
Merge150	0,00344	0,0551	0,02251	0,01099	0,0099	4,87E-04
Merge155	0,0798	0,00698	0,00763	0,00655	0,00368	0,00364
Merge2	0,11112	0,00953	3,16E-04	0,01323	0,02646	0,00834
Merge38	1,77E-04	0,03041	0,00544	0,06879	0,00942	0,02926
Merge66	0,00661	0,05525	0,0083	0,00406	0,00894	0,01668
Merge8	0,02003	3,16E-04	0,06256	0,00519	0,01734	0,01583
Merge85	0,00735	0,06775	0,06277	0,0209	0,01471	6,61E-04
oPt-0121	0,00292	6,56E-04	0,06415	2,87E-04	0,00814	0,0034
oPt-0246	0,02423	0,00224	0,0271	0,03924	0,0032	0,02958
oPt-0657	0,06941	0,00115	0,00634	0,02275	0,0032	4,52E-04
oPt-0802	0,02542	0,01775	0,01951	0,0762	0,02061	0,0012
oPt-10734	0,00323	0,01478	0,00264	0,07742	0,03053	0,03916
oPt-12924	0,0392	0,01595	0,05393	0,00753	0,02552	0,04111
oPt-13101	2,26E-05	0,00557	0,00285	0,00679	0,00144	0,00244
oPt-14113	0,06936	0,01168	0,00399	0,01876	9,19E-04	0,00541
oPt-14877	0,01471	0,00162	0,06891	0,00373	0,00452	0,01266
oPt-17060	0,00959	0,002	0,04865	0,00565	0,03047	0,0108
oPt-17855	0,0108	0,00651	0,01791	0,02541	0,04882	0,00356
oPt-3038	0,00301	0,00757	0,10786	0,0159	0,00419	0,01705
oPt-3145	6,34E-04	0,01255	0,03235	0,0175	0,01607	0,03695
oPt-3224	0,05428	0,02058	0,03759	0,00573	0,00526	0,00317
oPt-3345	0,01179	0,07166	0,00977	0,01397	0,00147	0,01987
oPt-3390	0,0703	0,01312	0,01561	0,00489	0,02106	0,00685
oPt-4136	0,00242	0,02558	0,04363	0,04161	7,48E-04	1,83E-04
oPt-4384	0,0084	0,01175	0,00509	0,04607	0,02092	0,00885
oPt-4396	0,05004	0,00647	0,01096	0,01403	0,0015	0,0226
oPt-5217	0,017	0,00931	0,01224	0,04535	0,03838	0,0358
oPt-5671	0,07638	0,00103	0,02237	0,00168	0,00371	0,02278
oPt-5986	0,02674	0,0146	0,01397	0,00246	0,0389	0,00315
oPt-7795	0,01037	0,01801	0,01318	0,06097	0,00339	0,01664
oPt-794463	0,22935	9,99E-04	2,96E-04	3,78E-04	0,00221	2,16E-05

oPt-8261	0,02781	0,03729	0,00202	0,05739	0,02379	0,02474
oPt-9348	0,00737	0,01418	0,06282	0,03709	0,01345	0,00948
oPt-9844	0,00473	0,00435	0,00717	0,00101	0,0582	0,09434
			**BIOMASS**			
Barb2-40	0,0102	0,02224	0,00605	0,07556	0,02579	0,01652
AM102	0,00544	6,84E-04	9,32E-04	0,02245	0,11231	0,00851
Merge121	0,01678	0,01227	1,25E-04	0,00654	0,00328	0,07654
Merge98	0,01432	0,04776	0,00763	0,01852	0,00674	0,01394
oPt-0121	0,00858	0,00189	0,04073	6,37E-04	0,00864	0,00297
oPt-10734	0,00312	0,05292	0,0247	0,03551	0,02546	0,01077
oPt-13064	0,007	3,78E-05	0,05981	0,00202	0,0081	0,00216
oPt-13088	0,02013	0,02453	0,04141	2,11E-04	0,02804	0,00607
oPt-14653	0,01587	0,00748	0,06682	0,00908	0,00289	0,00964
oPt-15938	0,01693	0,00223	0,0056	0,04573	0,01665	0,02674
oPt-18018	0,02429	0,01789	0,00171	0,05642	0,02267	0,00376
oPt-3029	0,00656	0,00861	0,06966	0,02567	0,00944	3,46E-04
oPt-3145	0,03164	0,00383	0,03923	0,00269	0,00172	0,02732
oPt-7946	0,07624	2,11E-04	0,006	0,00629	0,01249	0,00532
oPt-9844	0,00465	0,02995	6,73E-04	7,95E-04	0,04034	0,08766
oPt-9936	0,01706	0,01547	0,07502	0,00935	0,0011	7,99E-05
			**HI**			
Barb2-40	0,08388	4,96E-04	0,02432	0,04155	0,00924	0,07113
AME176	0,00378	0,01694	0,01193	0,02709	0,00506	0,02109
AME105	0,03828	0,00694	0,00206	1,53E-04	0,0094	0,01875
HvXan	5,32E-04	0,11209	2,46E-04	3,64E-04	0,00254	3,50E-05
AM30	6,03E-05	3,16E-05	6,15E-05	2,22E-04	0,00142	0,03957
AM07	0,02184	0,05347	0,0017	4,02E-04	0,00808	5,07E-04
MAMA07	0,00418	0,00264	0,05462	1,13E-08	0,07264	0,0547
Merge132	0,00941	0,03235	0,00683	0,01364	0,00722	0,02769
Merge149	8,13E-04	0,11349	3,41E-05	0,00134	0,00731	0,00512
Merge150	0,01867	0,07637	0,02573	0,01071	0,0035	0,00539
Merge2	0,08067	0,01145	0,00199	0,00161	0,03097	0,0225
Merge3	0,06495	0,00833	0,00564	0,00162	0,01895	0,02766
Merge59	0,00519	0,00141	0,01546	0,02313	0,00303	0,00288
Merge66	0,00195	0,09795	0,00751	0,00851	0,00825	0,00554
oPt-0802	0,06632	0,00203	0,00446	0,04143	0,0035	0,02164
oPt-0894	0,08781	0,00771	0,06392	0,01435	0,01121	0,0119
oPt-10036	0,03362	0,01358	0,00954	0,02061	0,06826	0,0797
oPt-14428	0,0034	0,01419	0,00147	0,00866	0,06399	0,02377
oPt-2211	0,06617	0,00859	0,00293	0,00454	0,00203	0,02195
oPt-3038	0,01262	0,00122	0,0606	0,01151	0,00397	0,02748
oPt-3413	0,06919	0,00195	0,00258	0,01292	0,00298	0,02972
oPt-388638	0,04482	0,01549	0,02386	0,0121	0,02646	0,02603
oPt-4156	0,08007	0,01274	0,00134	0,00433	0,01426	0,0252
oPt-5217	0,01947	0,00249	0,02133	0,00587	0,01619	0,02722
oPt-5521	0,00145	0,02273	0,0068	0,03519	0,01026	0,02476

oPt-5671	0,04557	9,37E-04	0,01822	0,00599	0,01958	0,01716
oPt-6446	0,00238	0,00198	0,00973	0,04356	0,0016	0,0011
oPt-6854	0,00987	0,02934	0,02256	0,00411	0,09659	9,75E-04
oPt-7483	3,34E-04	0,01919	6,80E-04	0,01674	0,02006	0,05089
oPt-793146	0,01237	0,00656	0,01014	0,00829	0,03638	0,00397
oPt-793467	0,07097	0,0117	8,23E-05	0,00863	0,01679	0,00919
oPt-795325	0,00419	0,05798	0,00335	0,00322	0,0113	0,0049
oPt-8261	0,05016	0,03499	0,00411	0,03611	0,0171	0,05614
			**GDD**			
HVM20	0,00532	0,01703	0,01227	0,01067	0,00582	0,01568
MAMA12	0,00687	0,01105	0,00167	0,01991	0,00225	0,00299
MAMA1	0,01258	0,00402	0,01145	0,06413	0,01459	0,0133
AM03	0,0125	0,00636	0,0719	0,00138	0,00159	0,00901
AM30	7,73E-05	0,01276	0,00431	3,28E-04	0,00381	0,00543
MAMA07	0,00366	0,01788	1,74E-05	0,0543	0,00468	0,01599
AM112	0,00315	0,05828	6,51E-04	0,00215	0,00132	0,00829
MAMA9	0,00903	0,00606	0,00849	2,38E-04	0,00762	0,00115
MAMA5	0,00767	8,13E-05	0,00415	0,01795	0,06496	6,28E-04
Merge24	0,02266	0,01231	0,01445	0,06476	0,07901	0,02789
Merge77	7,76E-04	0,00372	0,05661	0,00463	0,01397	0,00764
Merge82	0,00181	0,02182	0,00695	0,00394	7,06E-05	0,0168
oPt-10359	0,0257	0,0482	9,22E-04	0,01697	0,00797	0,01128
oPt-10891	0,02759	0,03251	0,01102	0,13036	0,08252	0,0329
oPt-13208	0,02472	0,00197	0,00608	0,00862	0,01581	0,0194
oPt-1340	0,02356	0,00767	0,00961	0,09447	0,06892	0,01812
oPt-16049	0,00887	0,01289	0,00136	0,00351	0,00686	3,24E-04
oPt-16902	0,00117	0,00904	0,051	0,01277	0,03827	0,02362
oPt-17035	0,00664	0,06965	0,00957	0,00256	8,84E-04	0,00339
oPt-18014	0,00606	0,07096	5,38E-04	0,00244	1,38E-04	0,01494
oPt-5519	0,00569	0,01912	0,02509	0,00573	0,04836	0,00155
oPt-7254	2,53E-05	0,00117	0,0656	0,01226	0,00171	0,00365
oPt-794388	5,72E-04	0,07701	0,00233	0,0013	0,00123	0,00392
oPt-794446	0,05848	0,01762	0,00243	0,0235	0,0371	9,67E-04
oPt-794556	0,00905	0,04688	0,00504	0,00555	0,0154	7,46E-04
			**RUST**			
Barb4-10	4,61E-05	0,06251	0,01161	0,00562	0,00315	0,00326
AM01	0,03494	6,92E-05	8,22E-04	0,00273	1,30E-04	0,01075
AM14	0,00486	2,37E-04	6,08E-04	0,02179	0,0019	8,77E-04
AM87	0,00247	0,05214	0,00645	8,40E-06	0,00259	0,00601
AM07	0,00645	0,00148	0,01262	0,00566	0,00469	0,05656
MAMA07	0,00415	0,01613	0,00351	9,60E-04	3,87E-04	0,00317
MAMA9	1,85E-04	9,34E-04	1,84E-04	0,00448	0,08614	0,0077
AM42	0,00134	0,00406	0,00101	3,11E-05	0,00129	0,0348
MAMA5	1,72E-05	0,00261	0,00698	0,00894	0,00257	0,00102
AM04	2,21E-06	0,02439	0,00661	7,19E-05	3,65E-04	0,00504
FESC12	0,03262	0,00122	7,95E-04	4,38E-04	0,03324	0,01003

Merge109	0,01322	0,0248	0,03857	0,00253	0,08238	0,0084
Merge11	0,01174	0,00378	0,00264	0,03352	0,00131	0,00457
Merge13	0,00615	0,03756	0,03477	2,27E-04	0,0094	0,00219
Merge136	0,00138	0,01634	0,01465	0,03155	7,62E-05	6,50E-04
Merge14	0,01736	0,09037	0,033	0,00171	0,00138	0,01432
Merge53	0,00966	0,01128	0,00613	0,00527	0,01883	0,01981
oPt-0332	9,77E-05	0,05426	0,00237	0,00148	0,0302	7,41E-04
oPt-10121	0,00987	0,03831	0,02097	0,00737	0,00718	0,03425
oPt-11231	6,12E-04	2,18E-04	4,59E-04	0,00338	0,00604	0,05661
oPt-11494	0,0173	0,0217	0,01023	0,01695	0,0182	0,02858
oPt-12121	7,03E-04	0,00682	0,00307	2,30E-04	0,00142	0,04613
oPt-12215	0,00242	0,03752	0,01377	5,26E-04	0,0096	0,03716
oPt-12898	0,06765	0,03699	0,01026	0,01181	0,00876	0,01132
oPt-13149	0,00749	0,03274	0,02153	0,00184	0,10099	8,66E-04
oPt-13565	0,00188	0,00121	0,00185	0,00205	0,00398	0,05926
oPt-14210	0,01699	0,00916	0,00832	0,00392	0,03273	0,03031
oPt-14830	0,00898	0,05516	0,02221	0,00138	0,00439	0,00534
oPt-17188	0,00438	0,00313	0,00523	0,00786	0,0164	0,05229
oPt-17501	0,02646	0,00242	0,01395	0,0215	0,02449	0,00501
oPt-3145	0,00471	0,01988	0,04399	0,00873	0,00365	0,00304
oPt-3484	0,00551	0,01055	0,02412	0,00486	0,01788	0,00729
oPt-388490	0,00139	0,04252	0,06695	0,00107	0,01989	0,0018
oPt-388855	0,01447	0,01286	0,02037	0,00545	0,01248	0,03136
oPt-4497	4,02E-04	0,03268	0,03802	1,64E-04	0,00722	0,00337
oPt-4502	0,03942	0,00447	0,00786	0,00302	0,00709	0,00379
oPt-4961	0,01016	0,00739	0,01661	0,00279	0,02705	0,05615
oPt-794289	0,00619	0,02922	0,02901	0,00436	0,01518	0,04264
oPt-794313	0,02543	0,04042	0,03787	0,00667	0,00573	0,02902
oPt-795337	0,03818	0,00136	0,01569	7,53E-04	0,00347	9,74E-04
oPt-9546	0,01076	0,03244	0,01463	0,03341	0,00107	0,00243
oPt-9895	0,01245	0,03996	0,01255	0,00144	0,01929	0,00823

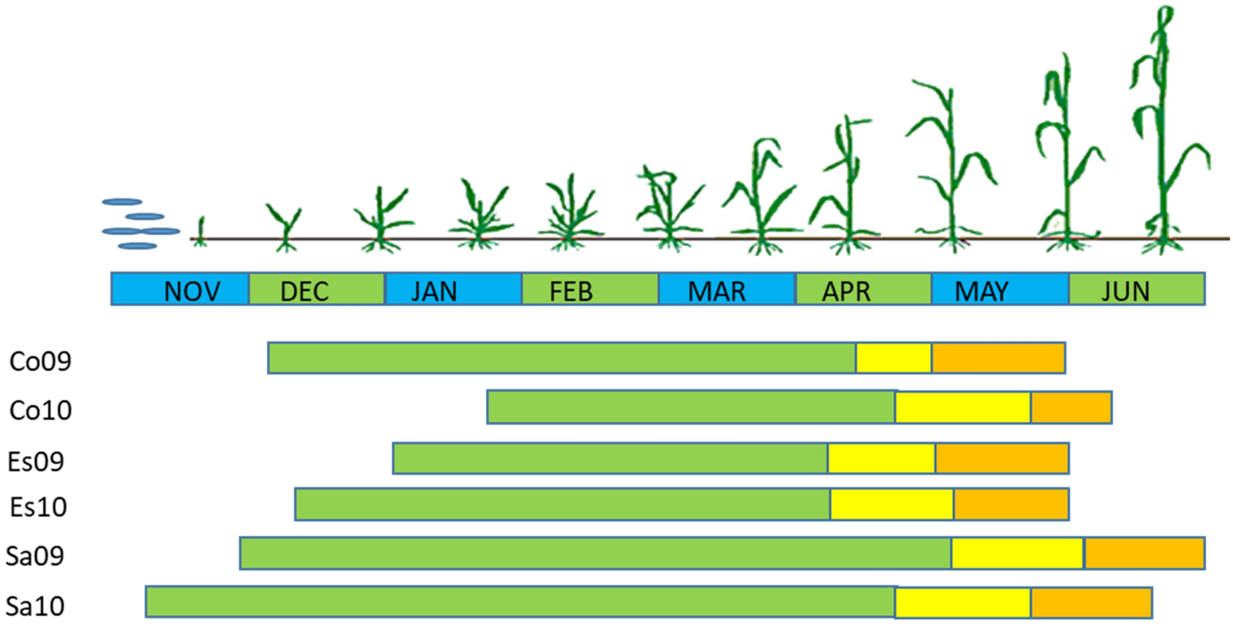

Supplemental Figure 1. Development of the oat trials in the different environments assessed, where green segments indicated pre-heading stage, yellow segments indicate heading period and orange segments indicate post-heading period.

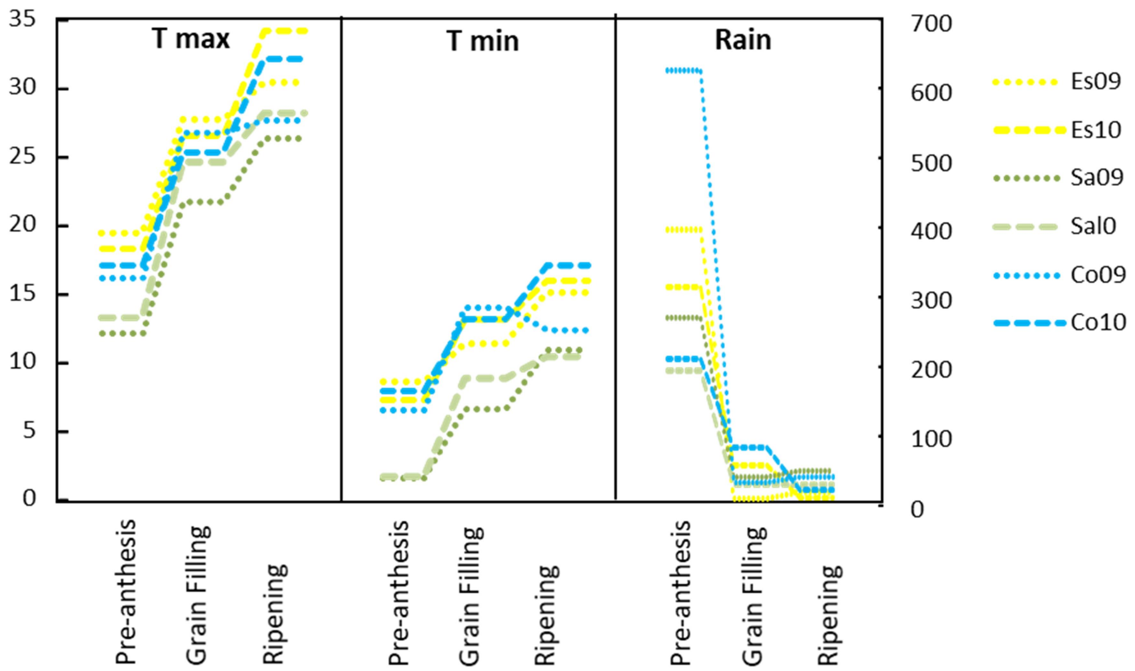

Supplemental Figure 2. Climatic variables including average maximun and minimum temperature (Tmax and Tmin, respectively) and cumulative rainfall (Rain) during pre-anthesis, grain filling and rippening period in the different Mediterranean environments studied: Es, Escacena; Co, Cordoba; and Sa, Salamanca during 2008-2009 (09) and 2009-2010 (10) seasons.

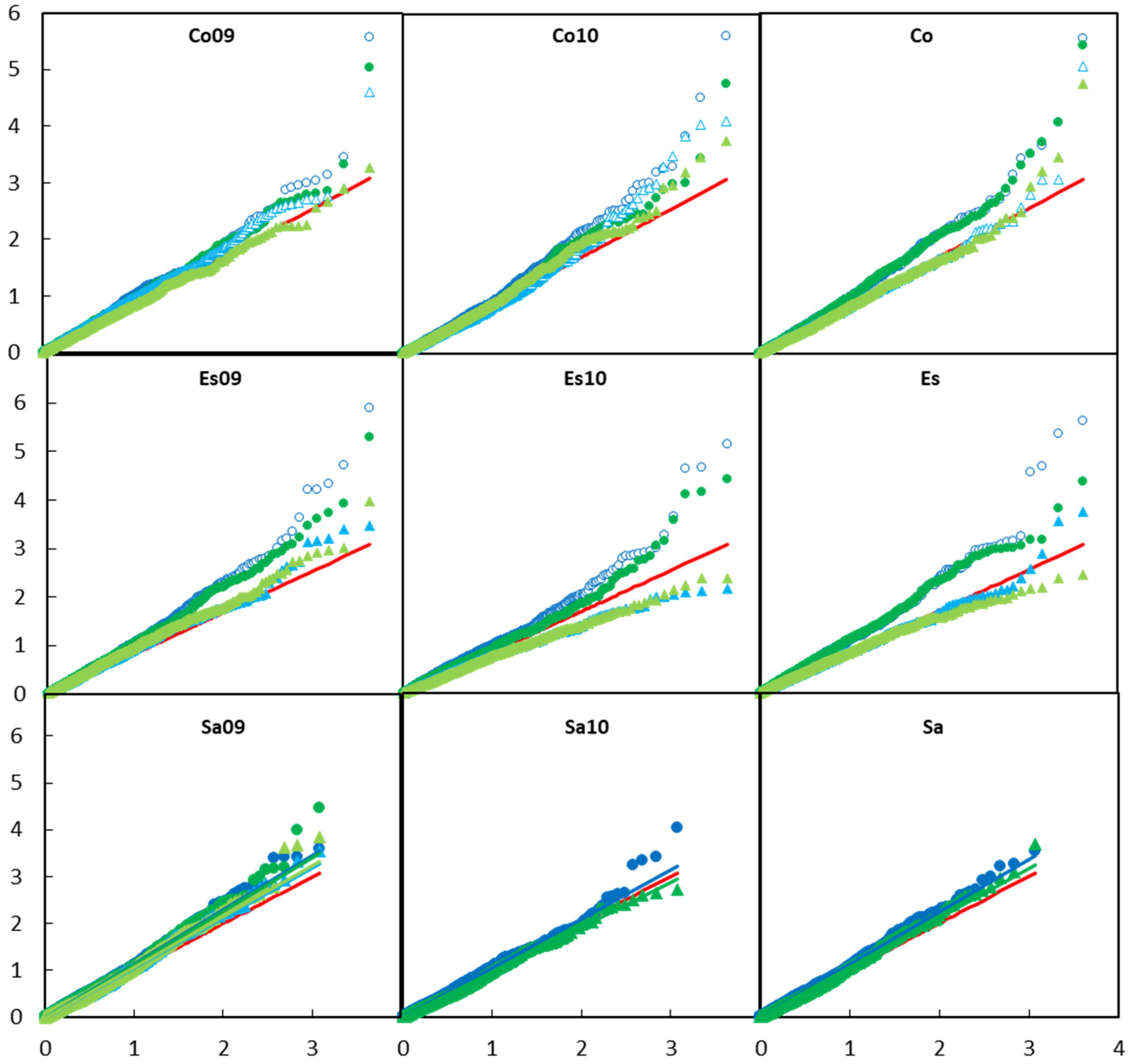

Supplemental Figure 3. Distribution of p values for the different models used in this study for the association between markers and rust severity in the localities tested: Cordoba (Co), Escacena (Es) and Salamanca (Sa) in 2009 (09) and 2010(10). Axes represented the expected p values versus the observed p values in the negative log10 scale where the solid line represent the null expectation (absence of type I error). Circles and triangles represent General linear models corrected for population structure and Mixed linear models, respectively, either using means data (blue symbols) or BLUPs (green symbols).

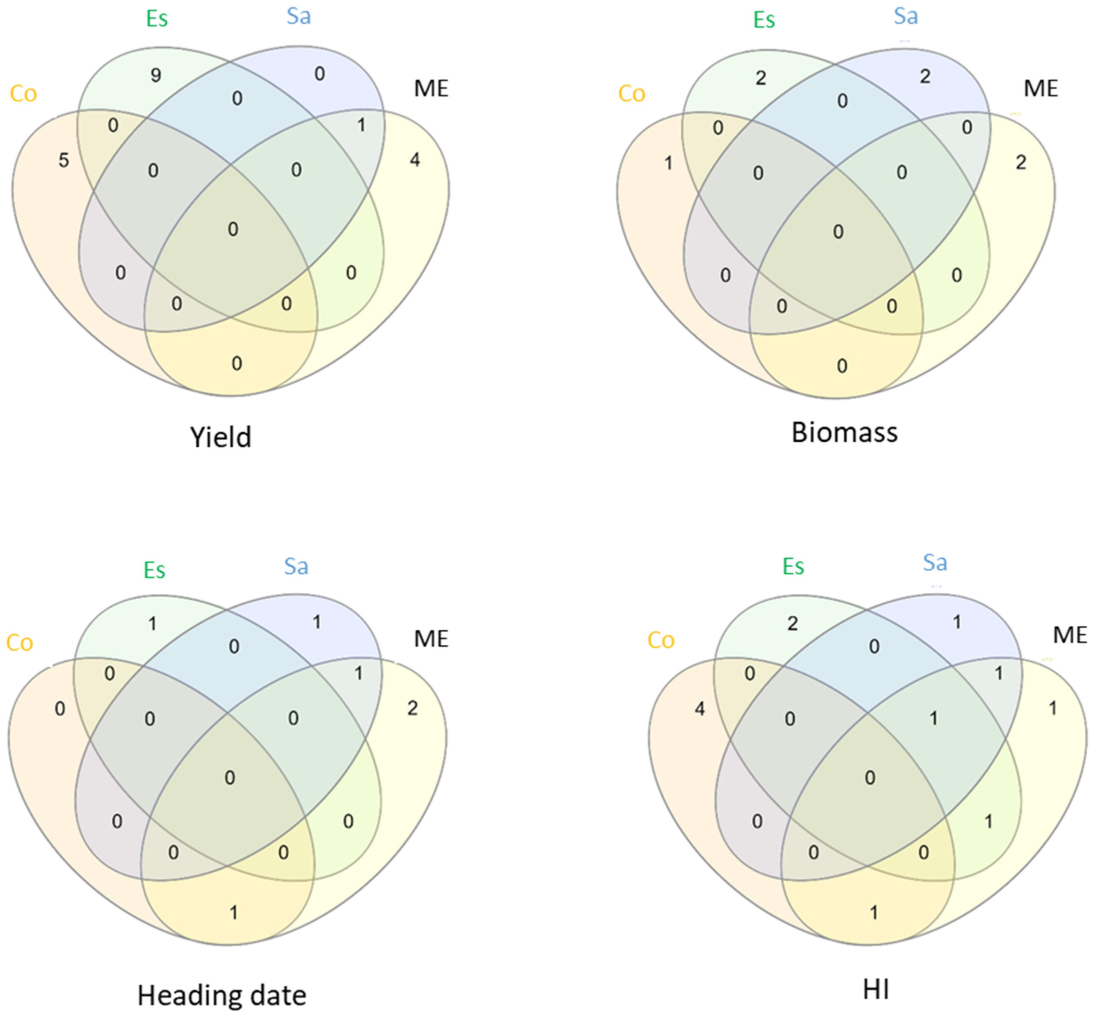

Supplemental Figure 4. Venn diagrams indicating the number of significant markers identified in each locality tested: Cordoba (Co), Escacena (Es) and Salamanca (Sa) and in the mega-environment (ME) for the different agronomic traits.
